# Development of serial X-ray fluorescence holography for radiation-sensitive protein crystals

**DOI:** 10.1107/S1600577522011833

**Published:** 2023-01-20

**Authors:** Artoni Kevin R. Ang, Yasufumi Umena, Ayana Sato-Tomita, Naoya Shibayama, Naohisa Happo, Riho Marumi, Yuta Yamamoto, Koji Kimura, Naomi Kawamura, Yu Takano, Tomohiro Matsushita, Yuji C. Sasaki, Jian-Ren Shen, Kouichi Hayashi

**Affiliations:** a Department of Physical Science and Engineering, Nagoya Institute of Technology, Gokiso, Showa, Nagoya 466-8555, Japan; b Synchrotron Radiation Research Center, Nagoya University, Furo, Chikusa, Nagoya 466-8603, Japan; c Division of Biophysics, Department of Physiology, Jichi Medical University, Yakushiji, Shimotsuke, Tochigi 329-0498, Japan; d Department of Computer and Network Engineering, Graduate School of Information Sciences, Hiroshima City University, Asa-Minami-ku, Hiroshima 731-3194, Japan; e Japan Synchrotron Radiation Research Institute (JASRI), Sayo, Hyôgo 679-5198, Japan; f Graduate School of Information Sciences, Hiroshima City University, Asa-Minami-ku, Hiroshima 731-3194, Japan; g Graduate School of Science and Technology, Nara Institute of Science and Technology, Ikoma 630-0192, Japan; h Graduate School of Frontier Sciences, University of Tokyo, Kashiwa, Chiba 277-8561, Japan; i Research Institute for Interdisciplinary Science and Graduate School of Natural Science and Technology, Okayama University, Tsushima Naka, Okayama 700-8530, Japan; University of Essex, United Kingdom

**Keywords:** X-ray fluorescence holography, atomic structures, protein structures

## Abstract

X-ray fluorescence holography (XFH) is a powerful atomic resolution technique capable of directly imaging the local atomic structure around atoms of a target element within a material. Although it is theoretically possible to use XFH to study the local structures of metal clusters in large protein crystals, the experiment has proven difficult to perform, especially on radiation-sensitive proteins. Here, the development of serial X-ray fluorescence holography to allow the direct recording of hologram patterns before the onset of radiation damage is reported. By combining a 2D hybrid detector and the serial data collection used in serial protein crystallography, the X-ray fluorescence hologram can be directly recorded in a fraction of the measurement time needed for conventional XFH measurements. This approach was demonstrated by obtaining the Mn *K*α hologram pattern from the protein crystal Photosystem II without any X-ray-induced reduction of the Mn clusters. Furthermore, a method to interpret the fluorescence patterns as real-space projections of the atoms surrounding the Mn emitters has been developed, where the surrounding atoms produce large dark dips along the emitter–scatterer bond directions. This new technique paves the way for future experiments on protein crystals that aim to clarify the local atomic structures of their functional metal clusters, and for other related XFH experiments such as valence-selective XFH or time-resolved XFH.

## Abstract 2

In this work, serial X-ray fluorescence holography is developed and its capabilities are demonstrated by obtaining hologram patterns from the protein crystal Photosystem II before any signs of radiation-induced damage. This new technique paves the way for future experiments on protein crystals that aim to clarify the local atomic structure of their functional metal clusters.

## 1. Introduction

Atomic resolution holography techniques, such as photoelectron holography, X-ray fluorescence holography and neutron holography, are a family of powerful experimental techniques that allow the direct probing of the local structures around atoms of a target element (Daimon, 2018 ▸, 2020 ▸). These techniques are capable of simultaneously recording the intensity and phase of the scattered beams, allowing the direct, model-free extraction of the 3D positions of the scatterer atoms around the emitter atoms.

X-ray fluorescence holography (XFH) (Tegze & Faigel, 1996 ▸; Faigel & Tegze, 1999 ▸; Hayashi *et al.*, 2012 ▸; Hayashi & Korecki, 2018 ▸), in particular, is a robust and powerful technique that has been used to reveal the local structures around active sites in various functional materials. In XFH, atoms of a target element are excited by an incident X-ray, which then emit fluorescent X-rays. These fluorescent X-rays act as a reference wave, which is then scattered by the surrounding atoms (object wave). The interference between the reference and object waves stores information about the 3D arrangement of the scatterers. This interference pattern can be treated as a hologram pattern, where the 3D arrangement of atoms can be directly reconstructed using Barton’s method (Barton, 1991 ▸), which is a Fourier transform based algorithm, or advanced reconstruction algorithms such as the L_1_-regularized linear regression method (Matsushita, 2018 ▸).

While almost all reported XFH experiments have been on inorganic functional materials, XFH is also expected to be capable of resolving the local structures around metal atoms in organic materials. This was first proposed back in 1996 (Fadley & Len, 1996 ▸); however, experimental difficulties in dealing with organic samples have significantly stalled progress in this field. These difficulties included low metal concentrations, large complicated unit cells, samples consisting mostly of light elements and the susceptibility of these samples to radiation damage. However, recent advances in synchrotron radiation facilities, X-ray detectors and cryogenic cooling have provided possible solutions to these challenges.

Work on adapting XFH for organic samples started recently, with the first bioXFH setup for protein crystal samples developed and tested on human hemoglobin (Hb) crystals in 2016 (Sato-Tomita *et al.*, 2016 ▸). The experimental difficulties were addressed by using large *P*4_1_2_1_2 Hb crystals, a toroidal graphite energy analyzer crystal to collect and focus the fluorescent X-rays, an N_2_ cold-gas flow system and the introduction of a new χ-circle stage and sample holder. While the experiment successfully prevented radiation damage to the protein crystal, the atomic reconstruction has not yet been fully interpreted. The resulting reconstructed atomic image is a complicated superposition of the 16 different Fe local environments, requiring more sophisticated methods of analysis. A more recent attempt on sperm whale myoglobin, a simpler crystal with the space group *P*2_1_, successfully obtained the reconstructed image (Sato-Tomita *et al.*, 2022 ▸). In this work, the atomic image around the Fe heme was reconstructed from the measured holograms, and some of the atomic images reflected the actual atomic positions.

Recently, clear atomic reconstruction was obtained and interpreted from XFH experiments on the organic superconductor κ-(BEDT-TTF)_2_Cu[N(CN)_2_]Br or κ-Br (Ang *et al.*, 2021 ▸). κ-Br is an organic charge-transfer salt that is widely studied in strongly correlated electron physics, where an Anderson-type metal–insulator transition is induced by the introduction of random defects by X-ray irradiation (Sano *et al.*, 2010 ▸). Atomic reconstructions from XFH, molecular dynamics simulations and hologram calculations have shown experimental evidence for the previously proposed ‘bond-shifted’ model (Kang *et al.*, 2017 ▸). Diffraction images and resistivity measurements before and after XFH experiments showed no additional radiation damage. Contrary to the complicated local structures around the Fe heme in hemoglobin or myoglobin, there are only four inequivalent Cu sites in κ-Br and they all lie in the anion layer, significantly simplifying the interpretation of the atomic reconstructions.

To date, all bioXFH experiments reported have been performed using the bioXFH apparatus, in which the angular dependence of the fluorescent X-rays is recorded as the sample is rotated point-by-point along the polar and azimuthal angles, taking several hours to record a full hologram pattern (Sato-Tomita *et al.*, 2016 ▸, 2022 ▸; Ang *et al.*, 2021 ▸). For more robust crystals like hemoglobin or myoglobin, cooling the sample to 100 K and controlling the photon flux enable collection of the hologram pattern before the onset of radiation-induced global damage. No changes in the diffraction patterns were observed after recording the holograms. However, at these dosage levels, specific damage around water molecules and active redox sites can still occur (Garman, 2010 ▸), and this bioXFH apparatus is no longer appropriate for these kinds of sensitive protein crystals.

**Figure 1:**
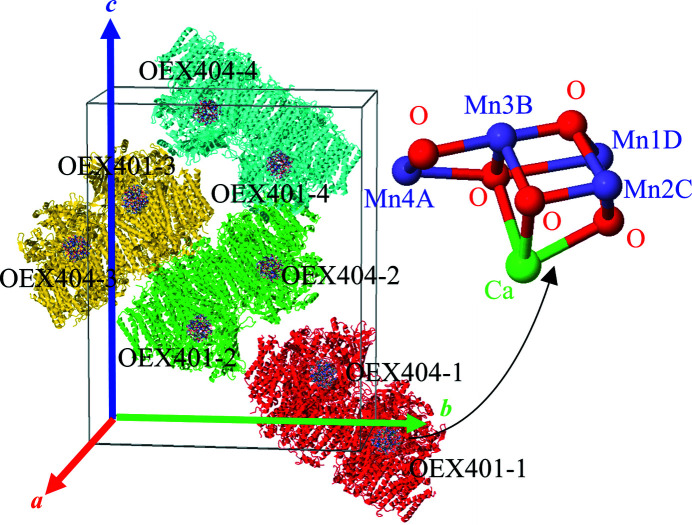
Crystal structure of PSII determined from protein X-ray crystallography (PDB entry 3wu2), with the relative positions of the Mn_4_CaO_5_ clusters highlighted. Inset: the Mn_4_CaO_5_ cluster.

An example of sensitive protein crystals is the protein membrane complex Photosystem II [PSII (Dau & Haumann, 2008 ▸; Shen, 2015 ▸)]. The oxygen-evolving complex (OEC) of PSII is the catalytic center of the photosynthetic oxidation of water by green plants. In this process, the OEC, which consists of an Mn_4_CaO_5_ cluster, cycles through a series of redox states as described by the Kok cycle, where these states are designated as S_*i*_ (where *i* = 0–4). The structure of PSII, and its OEC, have been extensively studied by synchrotron X-ray diffraction (XRD) with gradually increasing resolution (Zouni *et al.*, 2001 ▸; Kamiya & Shen, 2003 ▸; Ferreira *et al.*, 2004 ▸; Guskov *et al.*, 2009 ▸; Umena *et al.*, 2011 ▸). The Mn_4_CaO_5_ cluster consists of a cubane-like structure, with one Ca and three Mn atoms (Mn1D, Mn2C, Mn3B) occupying four corners, and O occupying the other four, while the last Mn atom (Mn4A) is connected to the cubane by two di-μ-oxo-bridges [Fig. 1 ▸]. Although the PSII structure obtained at 1.9 Å resolution provided remarkable details (Umena *et al.*, 2011 ▸), the average Mn–ligand and Mn–Mn distances were slightly longer than those extracted from extended X-ray absorption fine structure [EXAFS (Yano, Pushkar *et al.*, 2005 ▸; Dau *et al.*, 2008 ▸; Glöckner *et al.*, 2013 ▸)]. Recently, the ‘low-dose’ structure of PSII was revealed using extremely low-dose synchrotron XRD (Tanaka *et al.*, 2017 ▸), and the ‘damage-free’ structure was obtained from a combination of large crystals and the femtosecond X-ray pulses of an X-ray free-electron laser (XFEL) (Suga *et al.*, 2015 ▸), which provided shorter Mn–Mn distances, consistent with those obtained from EXAFS studies. The valence of each Mn ion in the Mn_4_CaO_5_ cluster of PSII S_1_ [2 × Mn(III), 2 × Mn(IV)] have been asserted based on the results of experiments such as X-ray absorption spectroscopy [XAS (Dau *et al.*, 2008 ▸; Glöckner *et al.*, 2013 ▸; Roelofs *et al.*, 1996 ▸; Yachandra *et al.*, 1996 ▸; Robblee *et al.*, 2001 ▸], Fourier transform infrared spectroscopy (Chu *et al.*, 2001 ▸, 2004 ▸; Debus *et al.*, 2005 ▸), various electron paramagnetic resonance (EPR) spectroscopy techniques (Kulik *et al.*, 2007 ▸; Cox *et al.*, 2011 ▸; Stich *et al.*, 2011 ▸; Asada *et al.*, 2013 ▸), through the analysis of the Jahn–Teller distortion effects from XRD studies (Suga *et al.*, 2015 ▸) and from theoretical calculations. These results suggest that the typical doses in protein crystallography experiments reduce, or partially reduce, the Mn ions and change the local structure around Mn, making the determination of the structural arrangement and the valence states of Mn difficult.

In this work, we develop a novel approach to determine the local structure of the metal clusters in protein crystals. By adapting XFH for low doses, the local structure of these metal clusters can be directly obtained. To minimize X-ray exposure of the crystals during the XFH measurements, the holograms can be directly imaged using a 2D hybrid pixel detector allowing much faster data acquisition. We have previously demonstrated the direct imaging of the Fe *K*α holograms of the mixed-valence compound magnetite [Fe_3_O_4_ (Ang *et al.*, 2018 ▸)]. Building from this work, the principles of serial crystallography were incorporated into the experiment to further reduce the X-ray exposure of the protein crystal samples. This new approach is demonstrated on large PSII S_1_ crystals, where the crystal structure and Mn valence states are already well established in the literature. By scanning the irradiation point across the surface of several PSII crystals, the holograms were directly imaged using a 2D hybrid pixel detector prior to the onset of X-ray-induced reduction of the metal clusters. By interpreting the holograms in terms of dips in the fluorescent X-ray intensity in the forward-scattering directions, real-space projections of the arrangement of the atoms in the Mn clusters were obtained. Furthermore, we attempted valence-sensitive XFH by tuning the incident X-ray energy based on the small shifts in the Mn *K*-edge of the different Mn ions. The real space projections in the Mn(III) and Mn(IV) holograms showed modest indications of the different local structure around these ions. These results demonstrate a new and straightforward approach for XFH experiments on highly sensitive protein crystals and the future possibility of simultaneous valence-selective XFH experiments on the metal clusters in protein crystals.

## 2. Material and methods

### 2.1. X-ray fluorescence holography measurements

**Figure 2:**
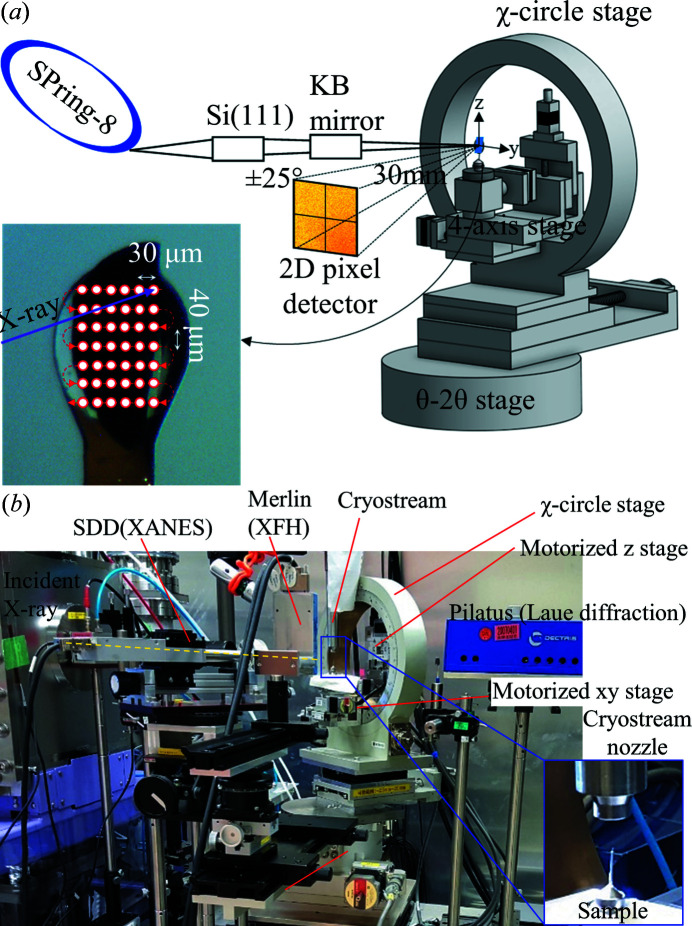
sXFH experiment to visualize the local structure around metal atoms in protein crystals at 100 K. (*a*) Schematic of the sXFH measurements. (*b*) Photograph of the experimental apparatus taken at BL39-XU, SPring-8. Reproduced with permission from The Japan Society of Applied Physics (Copyright 2020) (Ang *et al.*, 2020 ▸).

Serial X-ray fluorescence holography (sXFH) experiments were performed on BL39XU of SPring-8, Japan, using X-rays focused by Kirkpatrick–Baez (KB) mirrors (Suzuki *et al.*, 2013 ▸). Using the KB mirrors and a variable Al film attenuator, the beam spot size and the X-ray photon flux were set to 7 µm × 10 µm and ∼2 × 10^9^ photons s^−1^, respectively. To perform sXFH experiments, the bioXFH apparatus previously described by Sato-Tomita *et al.* (2016 ▸) was modified to allow the scanning of the irradiation point, and the use of a 2D X-ray detector. A schematic of the sXFH apparatus is shown in Fig. 2 ▸.

The sXFH apparatus consists of a precision motorized four-axis (*YZ*-swivel-tilt) sample stage that is mounted on the χ-circle stage of the bioXFH apparatus (Sato-Tomita *et al.*, 2016 ▸). This entire assembly is mounted on a 2θ goniometer, where the sample stage assembly and the detector assembly can be rotated independently.

The samples are cooled to 100 K using a liquid-nitro­gen gas-flow system (Cryostream 800, Oxford Cryosystems, Inc.) with the cryostream nozzle set according to the protocols for cryogenic X-ray crystallography.

The scanning of the irradiation point necessary for these experiments is incompatible with inverse-mode XFH, where the sample is rotated point-by-point along the θ axis (0–75°) and ϕ axis (0–360°) as the fluorescent X-ray is recorded. In normal-mode XFH, the hologram can be directly imaged with a hybrid pixel detector (Ang *et al.*, 2018 ▸; Bortel *et al.*, 2019 ▸), allowing faster data acquisition.

To record the holograms, a Medipix3-based quad chip version of the Merlin 2D detector system (Quantum Detectors) was used (Plackett *et al.*, 2013 ▸; Ballabriga *et al.*, 2018 ▸). This is the same detector used in a previous valence-sensitive normal-mode XFH experiment (Ang *et al.*, 2018 ▸), and in a related experiment on Kossel lines (Bortel *et al.*, 2016 ▸; Faigel *et al.*, 2016 ▸). The detector features high spatial resolution and dynamic range (256 × 256 pixels × 24 bit at 110 µm pixel size), which makes it suitable for holography experiments. The much larger EIGER X 1M 2D detector has also been used in a normal-mode XFH apparatus optimized for speed, where statistically relevant Ni holograms from an NiO crystal were recorded in a single image taken in 1 s (Bortel *et al.*, 2019 ▸). However, the Merlin quad chip detector allows two simultaneous energy thresholds to measure photons in a narrow-energy-window mode, which allows the user to record element-selective holograms from more complicated samples.

In this setup, the Merlin detector was set 30 mm from the sample. A Cr filter (4 µm film on a 8 µm Kapton sheet) was placed in front of the detector array to block the incident X-rays. Additional shielding was also set in front of the detector to prevent unwanted scattering reaching the detector.

### 2.2. Data collection

Each PSII crystal is scanned along the *y* and *z* directions as indicated in Fig. 2 ▸(*a*), with step sizes of 30 µm and 40 µm, respectively, to distribute the dosage and minimize radiation damage (Yano, Kern *et al.*, 2005 ▸). Images of the fluorescence intensity patterns were recorded at each point at an integration time of 10 s, resulting in an average dose of 0.15 MGy per point, as calculated using the program *RADDOSE* (Zeldin *et al.*, 2013 ▸). Table 1 ▸ summarizes the experimental conditions of the sXFH measurements on PSII.

**Figure 3:**
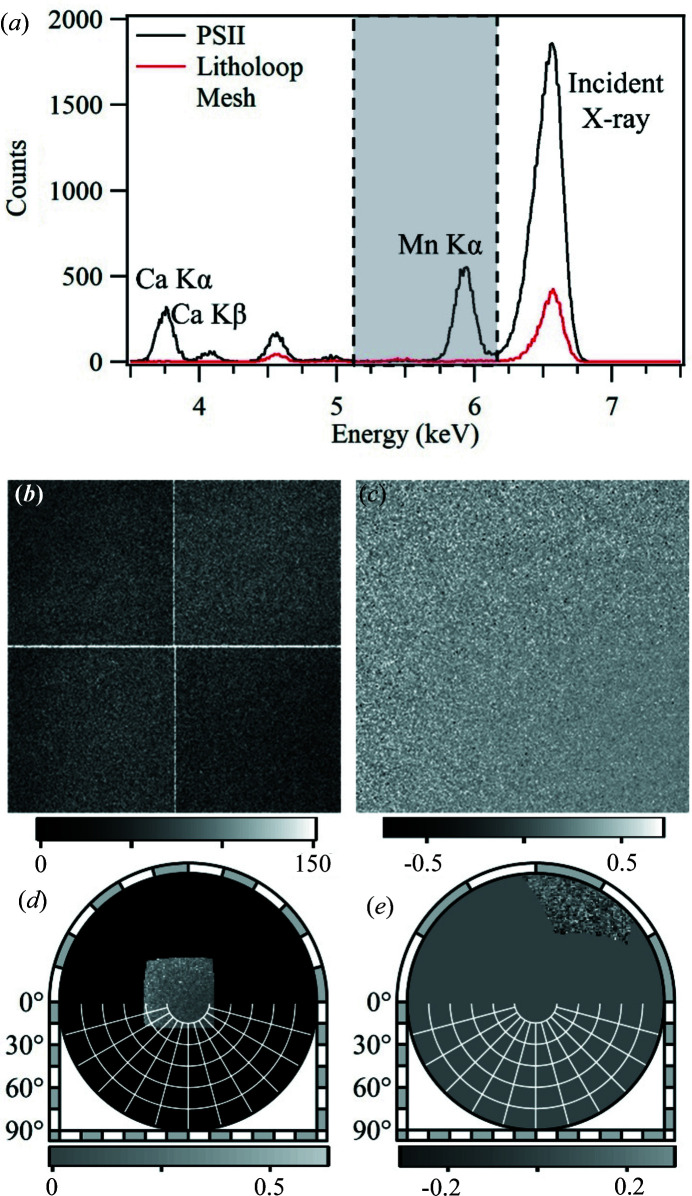
(*a*) Typical fluorescence spectra from a PSII crystal showing the Mn *K*α and Ca *K*α peaks, and from an empty Litholoop mesh taken at an incident X-ray energy of 6.565 keV. A typical Mn *K*α hologram image from a PSII crystal at different stages of data processing: (*b*) the total image from a single crystal, (*c*) normalized by dividing with background image, (*d*) after converting to a spherical projection, and (*e*) after flattening and rotating the pattern to the correct orientation.

The fluorescence intensity patterns were recorded using the 24 bit differential mode of the Merlin detector, with two simultaneous energy thresholds set at 5.15 keV and 6.15 keV. The fluorescence spectra at the detector position were confirmed with an SDD detector (XR-100SDD, Amptek Co. Ltd). A typical fluorescence spectrum from a PSII crystal, taken at an incident X-ray energy of 6.565 keV, is shown in Fig. 3 ▸(*a*), where the Mn *K*α and Ca *K*α peaks and a peak from the incident X-ray can be clearly observed. The Ca *K*α peak contains signals from both the Ca atoms in the OEC of PSII and the Ca atoms in the cryoprotectant. An additional fluorescence peak at ∼4.5 keV was also detected and was attributed to the Litholoop. Aside from the energy-windowing from the simultaneous energy thresholds, a Cr thin-film filter was also placed in front of the detector to block the scattered incident X-rays.

### 2.3. Hologram data processing

Before data processing, the raw images (energy-windowed 5.15–6.15 keV) obtained from each PSII crystal are first integrated, and any hot or dead pixels are removed using a 3 × 3 median filter. Figs. 3 ▸(*b*)–3(*e*) show a typical hologram pattern obtained from a PSII crystal at different stages of data processing.

To extract the hologram pattern from the fluorescence images, several data processing steps are needed. These are described in more detail by Ang *et al.* (2018 ▸); Bortel *et al.* (2019 ▸); and Matsushita, Muro, Matsui *et al.* (2020 ▸). First, all the images taken while scanning the irradiation point from each sample are integrated [Fig. 3 ▸(*b*)]. Then, each image (*I*_*n*_) is normalized using a normalizing pattern (*P*), obtained by integrating several images taken as the sample is slowly rotated in-plane. By rotating the sample, the holographic signal is averaged out in *P*, and only the low-frequency fluorescence background attributed to the sample–detector geometry remain. By normalizing each image pixel-by-pixel, *H*_*n*_ = *I*_*n*_/*P* [Fig. 3 ▸(*c*)]; this low-frequency background and any inhomogeneity in the sensitivity of the detector pixels can be removed. An additional histogram filter was applied to reduce noise.

The images *H*_*n*_, which are gnomonic projections, 2D projections of the spherical hologram pattern on the detector surface, are converted to a spherical projection [Fig. 3 ▸(*d*)]. To further remove low-frequency fluorescence background signals, the images are flattened by applying a Gaussian convolution on the images (*G*_*n*_), and dividing the original image with the background χ_*n*_ = *H*_*n*_/*G*_*n*_. The images are then rotated based on their orientations, as determined from the indexing of the diffraction images obtained from each sample. At this point, the fluorescence intensity oscillates around 1. The final hologram fragments from the different samples are composed into one hologram χ. Finally, a constant 1 is subtracted, and a low pass filter (σ = 10°) is applied.

To expand the recorded hologram in *k*-space, symmetry operations were applied based on the symmetry of the PSII S_1_ crystal.

### 2.4. Hologram pattern simulations

The intensity of the X-ray fluorescence holograms, χ(*k*), is expressed as

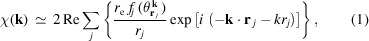

where *r*_e_ is the free electron radius, *f*_*j*_ is the atomic structure factor of the *j*th atom, and θ_*rj*_ is the angle between *k* and *r*_*j*_. For organic materials, however, the dynamic fluctuations caused by thermal vibrations are stronger than in inorganic materials, making it necessary to consider the effects of thermal vibrations. Thermal vibrations can be considered as atoms oscillating around their ideal positions with a Gaussian distribution with a standard deviation, σ, given as 〈*u* ^2^〉/6 = σ^2^/2. This introduces an additional term in the holographic oscillation (Matsushita, Muro, Yokoya *et al.*, 2020 ▸),

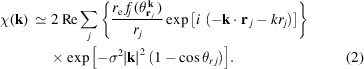

To simplify the calculations and reduce the computational times, the holograms were calculated using clusters constructed from only the Mn, O and Ca atoms within the OEC of PSII, based on atomic positions in the PDB entry 3wu2. For the total Mn *K*α holograms, 32 clusters, each centered on an Mn emitter atom, were created and used for the calculations. The angular positions (θ, ϕ) of the forward scattering (FS) dips for all Mn and Ca scatterer atoms from each Mn emitter atom were also calculated using the atomic positions in 3wu2 and are indicated by the purple and green circles overlaid on the hologram patterns.

### 2.5. Sample purification and crystallization

For the XFH experiments, the holograms were measured from many large isomorphous PSII crystals. The XFH signal from the sample was weak due to the low-dose irradiation that was necessary to avoid the X-ray reduction of the Mn atoms in the Mn_4_CaO_5_ cluster as much as possible. The samples were extracted from the thermophilic cyano­bacteria, *Thermo­synechococcus vulcanus*, and the PSII crystals were prepared based on the methods reported by Kawakami & Shen (2018 ▸).

To maintain the isomorphism between PSII crystals, the crystals were replaced with different concentrations of the cryo-protectant solutions step-by-step using a gentle dialysis method. The post-crystallization process using dialysis membrane (MWCO6000-8000 purchased from Spectra/Por dialysis) with a molecular weight cutoff of 6–8 kDa pore size was divided into six steps, in which each step was treated every hour, from the first crystal solution containing 10% PEG3000 to the final cryo-protectant solution containing 25% PEG3000 (polyethlyne glycol) and 20% di­methyl sulfoxide (DMSO). When a PSII crystal is frozen by flash-cooling, excess cryo-protectant solution covering the surface of a PSII crystal must be excluded, as this causes the attenuation of X-ray fluorescence. Therefore, the PSII crystal was placed on a mesh loop of diameter 400 µm or 1000 µm with 40 µm × 40 µm spacing, and the excess solution was absorbed from the back side by filter paper before flash-cooling the PSII crystal. To obtain different sections of the hologram pattern, PSII crystals oriented in different directions were prepared.

### 2.6. Evaluation of radiation damage

To evaluate the radiation damage introduced into the samples during the XFH experiments, the XANES spectra taken before and after the XFH scans are compared. The ‘before’ spectrum was obtained at a point on the surface of the sample outside the XFH scan region, and another spectrum was obtained from the center of the sample after the XFH scans with 1 eV steps and 2 s integration time. The Mn *K*-edge inflection point is determined from the zero point in the second derivative.

### 2.7. Determination of crystal orientation

The orientation of each PSII crystal relative to the Merlin detector was obtained from the indexing of the diffraction images taken by the Pilatus 100 K. Diffraction images were taken at a wavelength of 1.89 Å and indexed using *XDS* (Kabsch, 2010 ▸).

## 3. Results

### 3.1. Serial X-ray fluorescence holography

**Figure 4:**
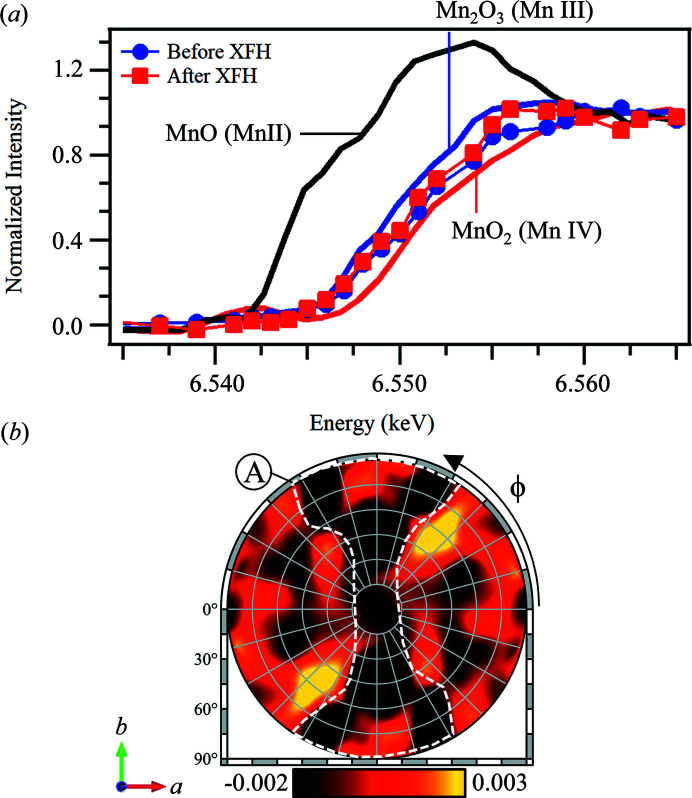
Mn *K*-edge absorption spectra and Mn *K*α holograms of PSII. (*a*) Mn absorption spectra of PSII taken before and after the sXFH experiment and various reference Mn oxide powders. (*b*) Mn *K*α hologram obtained at an incident X-ray energy of 6.565 keV (χ_H_).

Fig. 4 ▸(*a*) shows typical Mn *K*-edge absorption spectra of a PSII S_1_ crystal taken before and after the XFH experiment, which shows no observable shift in the Mn *K*-edge. The inflection points (zero point of the second derivative) at 6.551 keV agree with the previously reported inflection points for the S_1_ state (Roelofs *et al.*, 1996 ▸; Robblee *et al.*, 2001 ▸). These results demonstrate that the measures taken to minimize radiation damage were sufficient to prevent radiation-induced reduction of the Mn ions in the OEC.

Fig. 4 ▸(*b*) shows the Mn *K*α hologram integrated from nine PSII crystals. The hologram pattern appears to be dominated by the FS dips of the holographic oscillations (black in the color scale). The application of the low pass filter, the larger thermal vibration in protein crystals and the mostly light elements surrounding the Mn_4_CaO_5_ cluster (water molecules and amino-acid residues) suggest that the hologram consists mostly of holographic signal from scatterers near the Mn emitters.

To investigate how the larger thermal vibrations in proteins affect the holograms, the holograms were calculated with and without root-mean-square displacements of the scatterers due to static positional fluctuations within the crystal and dynamic fluctuations from thermal vibrations. The root-mean-square displacements were introduced into the hologram calculation by assuming isotropic vibrations of the scatterer atoms relative to the fixed emitter (〈*u* ^2^〉_Rel_), which is represented by a Gaussian distribution with a standard deviation σ.

**Figure 5:**
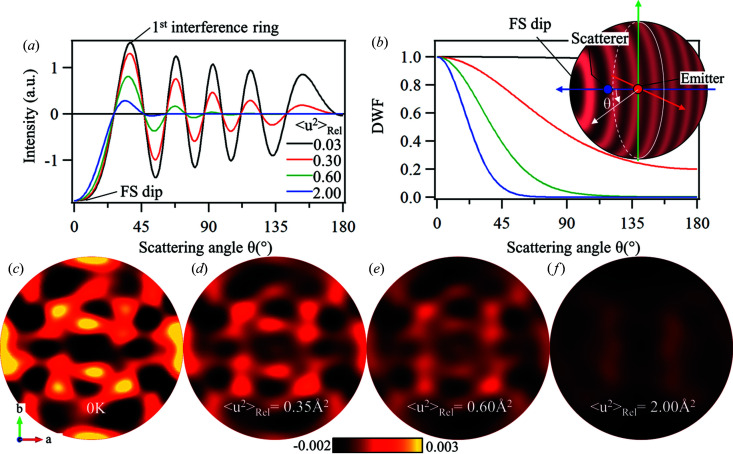
Evolution of XFH patterns with increasing root-mean-square displacements. (*a*) Holographic oscillations as a function of scattering angle simulated from a simple Mn–Mn dimer at different relative root-mean-square displacement values, χ_H_, and (*b*) their corresponding damping factors. Inset: spherical projection of the hologram calculated from the Mn–Mn dimer. (*c*)–(*f*) Evolution of the Mn *K*α holograms calculated from the OEC of PSII using different χ_H_ values: (*c*) Mn *K*α holograms simulated without thermal vibrations, and those with χ_H_ = 0.35 Å^2^ (*d*), χ_H_ = 0.60 Å^2^ (*e*) and χ_H_ = 2.00 Å^2^ (*f*).

First, Fig. 5 ▸(*a*) shows the holographic oscillations calculated from a simple Mn–Mn dimer system using different relative root-mean-square displacement values, 〈*u* ^2^〉_Rel_, whereas Fig. 5 ▸(*b*) shows the corresponding Debye–Waller factor or damping term. The holograms are composed of minima in the FS direction, surrounded by concentric higher-order interference rings, as shown in the inset of Fig. 5 ▸(*b*). As the 〈*u* ^2^〉_Rel_ values increase, the backscattering is suppressed and the FS dips become the prominent features of the hologram patterns.

Next, the Mn *K*α holograms of PSII were calculated. Since PSII has a large unit cell (122.2 Å × 228.5 Å × 286.4 Å) and consists mostly of light elements, only the atoms within the Mn_4_CaO_5_ cluster are used for the calculations. The atomic positions were extracted from the PDB entry 3wu2 (Umena *et al.*, 2011 ▸). The total calculated hologram is the superposition of the hologram patterns calculated for each of the 32 Mn atoms in a PSII unit cell. Each hologram was calculated using the Mn, Ca and O atoms located within the same Mn_4_CaO_5_ cluster as the emitter Mn (Fig. 1 ▸).

Fig. 5 ▸(*c*) shows the Mn *K*α hologram of PSII calculated without any thermal vibrations. With increasing 〈*u* ^2^〉_Rel_ values, the similarities with the experiment become more apparent. In Figs. 5 ▸(*d*)–5(*f*), a series of holograms were calculated with different 〈*u* ^2^〉_Rel_ values. As the 〈*u* ^2^〉_Rel_ value increases, the positive signals (bright yellow) in the hologram patterns are suppressed, and in Fig. 5 ▸(*f*), with an extreme 〈*u* ^2^〉_Rel_ = 2.00 Å^2^, the pattern is reduced to a large dark feature that bears strong resemblance to feature A in Fig. 4 ▸(*b*).

**Figure 6:**
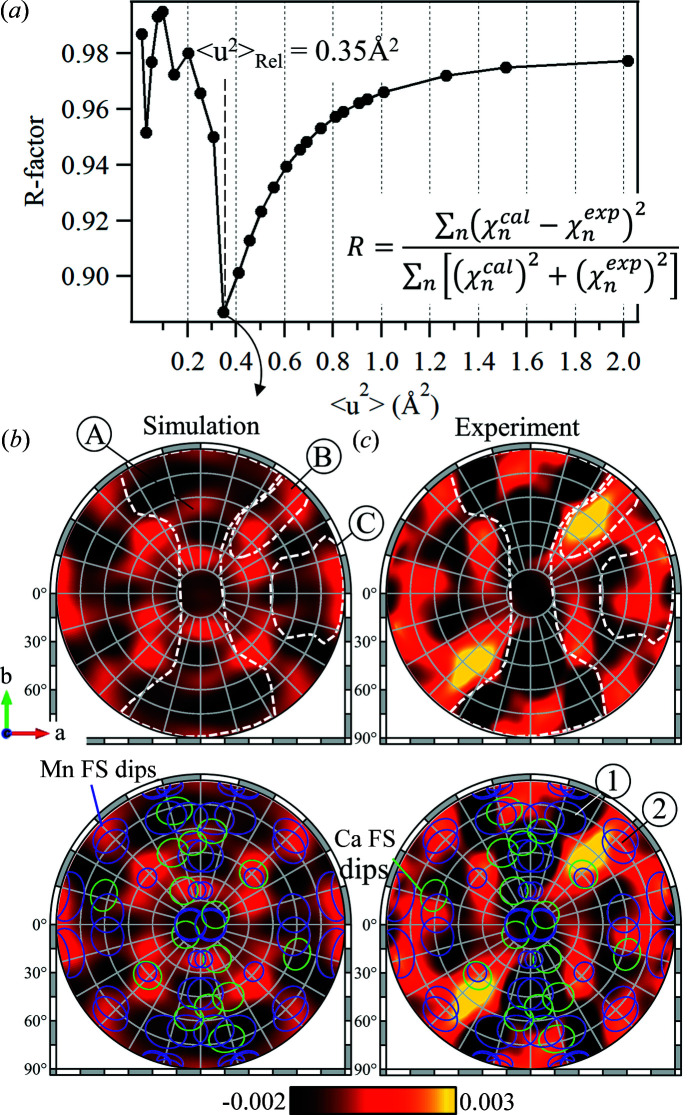
*R*-factor analysis between the calculated and experimental hologram patterns. (*a*) *R*-factor as a function of the relative mean-square displacements, 〈*u* ^2^〉_Rel_, of the scatterer atoms used in the Mn *K*α hologram calculations. (*b*) Calculated hologram with 〈*u* ^2^〉_Rel_ = 0.35 Å^2^ and (*c*) the experimental hologram. The dashed white lines are visual guides for features discussed in the text. Circular marks indicate the FS directions of Mn (purple) and Ca (green) scatterers calculated from each Mn emitter from PDB entry 3wu2.

To determine the best 〈*u* ^2^〉_Rel_ value, an *R*-factor analysis is performed (Kuznetsov *et al.*, 2014 ▸). The *R*-factor describes the agreement between the calculated and experimental hologram patterns, where a smaller *R*-factor means a better agreement. Fig. 6 ▸(*a*) shows the dependence of the *R*-factor on 〈*u* ^2^〉_Rel_. The minimization of the *R*-factor shows that the hologram calculated with 〈*u* ^2^〉_Rel_ = 0.35 Å^2^ shows the best agreement with the experiment. This calculated hologram pattern is shown in Fig. 6 ▸(*b*) and the experimental hologram is shown again in Fig. 6 ▸(*c*) for comparison and discussion.

There are several key features in the calculated hologram that are also clearly observed in the experimental hologram [highlighted by the dashed white lines in Figs. 6 ▸(*b*) and 6 ▸(*c*)]: region A, the large, dark and dog-bone-shaped region; region B, the bright feature observed at an azimuthal angle of 45°; and region C, a combination of dark and bright features.

For further interpretation of these features, the FS directions of all Mn and Ca scatterers were calculated from each Mn emitter and are superimposed on the lower images of Figs. 6 ▸(*b*) and 6 ▸(*c*). The angular positions of the circles on the images indicate the angular directions of the FS dips, and the relative sizes of the circles indicate the relative distances of the scatterer from the Mn emitters. These FS dips can be thought of as real-space projections of the positions of the scatterers around the emitter.

The origin of region A becomes apparent from the superimposed FS directions, where a dense collection of FS directions appear over the dark regions of the experimental hologram pattern in Fig. 6 ▸(*c*). Among these is the FS direction marked as (1) in Fig. 6 ▸(*c*), where a dark dip in the hologram pattern is observed in both the experiment and the calculation. This FS direction corresponds to an Mn4A–Mn3B emitter–scatterer pair where the Mn–Mn distance is 2.89 Å. The short emitter–scatter distance will result in a lower-frequency holographic signal and a larger FS dip (amplitude and width).

On the other hand, some FS directions appear as bright signals in both the experiment and the calculation, an example of which is the FS dip marked as (2). This occurs when the FS dip interferes with the first interference ring of an adjacent FS dip.

## 4. Discussion

The good agreement of the experimental results with the calculated hologram and calculated FS directions in Figs. 6 ▸(*b*) and 6 ▸(*c*) is a strong indication that the projection pattern obtained using sXFH contains structural information about the Mn_4_CaO_5_ cluster of PSII. Furthermore, the fact that the data were collected before any shifts in the Mn absorption spectra shows the tremendous potential of this method in studying the local structures of metal clusters in proteins before any radiation-induced damage.

In the current study, we interpreted the hologram patterns as real-space projections of the local structure around the emitter by analyzing the FS dips. This is similar to the tomographic interpretation of the directional fine structure in the absorption of white X-rays (Korecki & Materlik, 2001 ▸; Korecki *et al.*, 2006 ▸, 2009 ▸), also later termed white XFH (Dul & Korecki, 2012 ▸; Dąbrowski *et al.*, 2013 ▸). By employing polychromatic X-rays, a decrease in the coherence length results in the suppression of the higher-order interference fringes in the higher scattering angles, while the FS dip, which is largely energy independent, remains relatively unchanged. In this work, the combination of the large positional fluctuations in the PSII crystal and the application of the low pass filter on the hologram pattern also results in the suppression of the higher-order interference fringes while leaving the FS dip unchanged.

Furthermore, the FS dip approach used in this study can be thought of as the X-ray fluorescence analog of X-ray photoelectron diffraction (XPD) (Kuznetsov *et al.*, 2014 ▸). In XPD, the local structure information is extracted by analyzing the forward-focusing peaks (FFP) in the angular distribution of the photoelectron intensity. Using photoelectrons with high kinetic energies allows the FFP to dominate the XPD patterns and a real-space projection of the scatterers around the emitter can be obtained. XPD has been extensively used in studying local structures in 2D layers, thin films and interfaces. Aside from the FS dips, other features such as Kossel lines (Bortel *et al.*, 2016 ▸; Faigel *et al.*, 2016 ▸) or X-ray standing wave lines that may also appear in the hologram can also be used to extract structural information about the sample.

To improve the accuracy of hologram calculations used in this study, the effects of the positional fluctuations of the scatterers were directly introduced into the calculations by a Debye–Waller factor or damping term [Fig. 5 ▸(*b*) and equation (2)]. Until now, the effects of atomic fluctuations have been introduced into the hologram calculations by introducing a distribution of the atomic positions in the atomic model used in the calculations. This can include random Gaussian distributions in the atomic positions (Hayashi *et al.*, 2014 ▸; Hosokawa *et al.*, 2013 ▸; Kimura *et al.*, 2020 ▸) or, alternatively, using atomic positions extracted from molecular dynamics simulations (Ang *et al.*, 2021 ▸). Although these are good approximations that can result in the adequate reproduction of the atomic reconstruction, using a DWF or damping term directly in the hologram calculation is a simpler and more direct way to calculate the hologram pattern.

In the hologram calculations in Fig. 6 ▸, a root-mean-square displacement value of χ_H_ = 0.35 Å^2^ was used. This value obtained from the *R*-factor analysis in Fig. 6 ▸(*a*) is relative and the average 〈*u* ^2^〉 of the atoms in the Mn_4_CaO_5_ cluster can be obtained using (Kimura *et al.*, 2020 ▸)

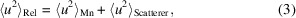

resulting in an average 〈*u* ^2^〉 = 0.29 Å^2^, or an average *B*-factor of 23.25 Å^2^ for all the atoms in Mn_4_CaO_5_. This is comparable with the average *R*-factors of the Mn atoms of the synchrotron radiation [*B*-factor ∼26 Å^2^ (Umena *et al.*, 2011 ▸)] or XFEL [*B*-factor ∼24 Å^2^ (Suga *et al.*, 2015 ▸)] structures, suggesting that, aside from dynamic fluctuations caused by thermal vibrations at 100 K, the atoms in the Mn_4_CaO_5_ cluster also have large positional fluctuations within the crystal. Though we were only able to extract average isotropic fluctuations in this work, we expect that in future work, the radial and angular positional fluctuations of Mn atoms can be extracted from more accurate hologram patterns, as this has already been demonstrated in XFH experiments on inorganic samples and organic crystals (Hosokawa *et al.*, 2013 ▸; Hayashi *et al.*, 2014 ▸; Ang *et al.*, 2021 ▸; Kizaki *et al.*, 2022 ▸). Once this is realized, valuable information to help understand the mechanisms of the oxidation process in PSII will be obtainable.

**Figure 7:**
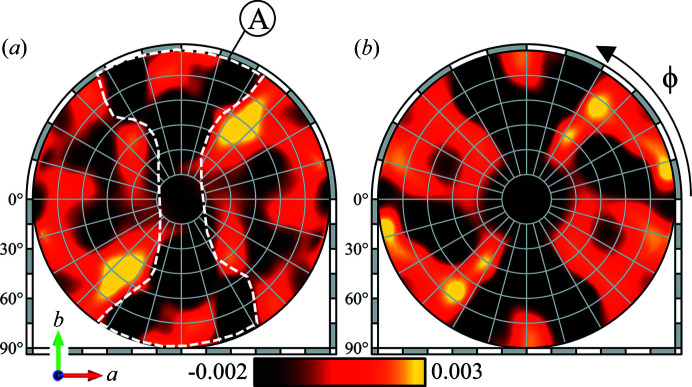
Mn *K*α hologram obtained at (*a*) a high incident X-ray energy of 6.565 keV (*χ*_H_) and (*b*) a low incident X-ray energy of 6.551 keV (*χ*_L_).

Aside from the Mn *K*α hologram obtained in Fig. 4 ▸(*b*), the experimental configuration will also allow valence-selective XFH. By carefully tuning the incident X-ray energy based on the small shifts in the Mn *K*-edge of the different Mn ions, valence-sensitive hologram patterns can be recorded. We performed preliminary valence-selective XFH experiments on the same PSII samples by recording an additional hologram pattern at 6.551 keV [below the Mn(IV) *K*-edge, but above the Mn(III) edge]. Fig. 7 ▸ shows the high (χ_H_) and low (χ_L_) energy Mn *K*α holograms taken at 6.565 keV and 6.551 keV, respectively. Both hologram patterns were taken from the same set of samples, and the only difference is the incident X-ray energy. The difference in the incident X-ray energies will result in a small change in the Mn(III):Mn(IV) contribution ratio, which means that, aside from small differences, the χ_H_ and χ_L_ holograms should be almost the same.

At 6.551 keV, the absorption and the subsequent fluorescence from Mn(IV) is suppressed, resulting in a hologram pattern that is mostly from the Mn(III) ions. The total hologram in Fig. 7 ▸(*a*), taken at an incident X-ray energy of 6.565 keV [above the Mn *K*-edge of both Mn(III) and Mn(IV)], *χ*_H_, is a superposition of hologram contributions from all Mn ions, χ_Mn(III)_ and χ_Mn(IV)_. Using the known Mn valence distribution of PSII S_1_ [2 Mn(III) and 2 Mn(IV) (Suga *et al.*, 2015 ▸)], the total hologram can be expressed as

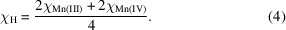

At the lower incident X-ray energy of 6.551 keV, both χ_Mn(III)_ and χ_Mn(IV)_ signals are suppressed due to the lower X-ray absorption, and the hologram *χ*_L_ can be expressed as

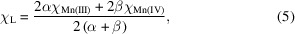

where the α and β parameters represent the decrease in the absorption spectra of χ_Mn(III)_ and χ_Mn(IV)_ relative to the absorption at 6.565 keV. From equations (3) and (4), χ_Mn(III)_ can be numerically extracted using

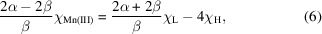

and subsequently χ_MnIV_ can be obtained using χ_H_, χ_Mn(III)_ and equation (4).

**Figure 8:**
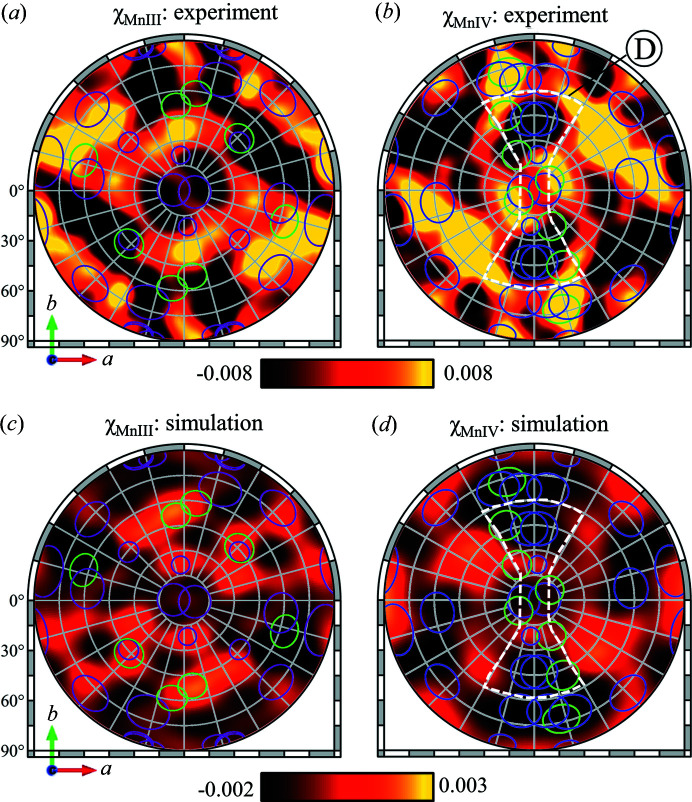
Valence-sensitive Mn *K*α hologram patterns χ_Mn(III)_ and χ_Mn(IV)_. (*a*) χ_Mn(III)_ and (*b*) χ_Mn(IV)_ hologram patterns extracted numerically from the experimental holograms using (α, β) = (0.75, 0.55) as determined from Mn absorption spectra. Calculated (*c*) χ_Mn(III)_ and (*d*) χ_Mn(IV)_ holograms with 〈*u* ^2^〉_Rel_ = 0.35 Å^2^. The FS directions of neighboring Mn and Ca atoms were calculated from either χ_Mn(III)_ (Mn1D and Mn4A sites) or χ_Mn(IV)_ (Mn2C and Mn3B sites) emitters and are indicated by the purple (Mn) or green (Ca) circles.

From the normalized intensity of the absorption spectra in Fig. 4 ▸(*a*), the α and β parameters are estimated to be 0.75 and 0.55, respectively. Using these parameters, the χ_Mn(III)_ and χ_Mn(IV)_ hologram patterns were extracted and are shown in Figs. 8 ▸(*a*) and 8 ▸(*b*).

To calculate the hologram patterns for Mn(III) or Mn(IV), only 16 clusters were used, with Mn emitters at the known sites of either the Mn(III) or Mn(IV) ions. Using the same χ_H_ parameter used for the total hologram pattern in Fig. 6 ▸(*b*), the hologram patterns were calculated for Mn(III) and Mn(IV) emitters in PSII and are shown in Figs. 8 ▸(*c*) and 8 ▸(*d*). The FS directions are once again superimposed on the holograms.

From the calculations of the hologram patterns and the FS directions, a distinct difference in the χ_Mn(III)_ and χ_Mn(IV)_ holograms can be observed: the large dark feature (region A) in χ_Mn(IV)_ that results from the dense arrangement of the FS directions. For the χ_Mn(III)_ hologram, there is no clear distinct feature in the hologram, the FS directions are evenly distributed.

This difference between χ_Mn(III)_ and χ_Mn(IV)_ can also be observed in the experimentally extracted holograms. The dark region in χ_Mn(IV)_, marked as region D in Fig. 8 ▸(*b*), is clearly observed in both the experimentally extracted and the calculated χ_Mn(IV)_ hologram patterns. On the other hand, the experimentally extracted χ_Mn(III)_, like the calculations, shows no distinct features. These results show that, while the agreement between the experiment and the calculations are not clear as shown in the the total hologram in Fig. 6 ▸, the distinct features in the hologram patterns can allow differentiation of the χ_Mn(III)_ and χ_Mn(IV)_ hologram patterns. In the case of the S_2_ state, where only one Mn(III) remains in the OEC, the distinction between χ_Mn(III)_ and χ_Mn(IV)_ will be clearer, making XFH analysis more straightforward. Thus, analysis of the χ_Mn(III)_ and χ_Mn(IV)_ hologram patterns can be an alternative approach to experimentally determining which Mn site is the remaining Mn(III).

In our experiment, a careful compromise had to be made between the total X-ray dose on the sample, the total photons collected by the 2D detector and the limited experiment times at synchrotron radiation facilities. Even under such limiting experimental conditions, we could already reach a certain agreement between the experimental and calculated holograms, as shown and discussed in the preceding sections. However, further optimization of the experiment would be necessary to obtain more accurate valence-selective holograms, because they must extract the small difference in the holograms taken at two different energies. By increasing the total photons collected during the experiment, either through longer exposure times, larger single-crystal samples or by measuring samples, the quality of the experimental hologram might be significantly improved. Alternatively, novel approaches to the extraction of the χ_MnIII_ and χ_MnIV_ holograms from the high- and low-energy holograms recorded in the experiment can also be developed. The subtraction in equation (6) can result in the propagation of uncertainty, and this can be seen in the higher contrast scale used in Fig. 8 ▸.

Several valence-selective XFH experiments have already been reported for inorganic crystals: a direct-imaging experiment using the same 2D hybrid pixel detector on magnetite (Fe_3_O_4_) (Ang *et al.*, 2018 ▸) and conventional XFH experiments using 0D detectors (avalanche photodiode or SDD) on yttrium oxide thin films (YO/Y_2_O_3_) and YbInCu_4_ (Stellhorn *et al.*, 2017 ▸; Hosokawa *et al.*, 2019 ▸, 2020 ▸). In the experiments on Fe_3_O_4_, the difference in the Kossel line features between the high- and low-energy hologram patterns clearly show the valence selectivity (Ang *et al.*, 2018 ▸). Atomic reconstructions of these valence-selective hologram patterns have resulted in mixed results. Ang *et al.* (2018 ▸) numerically extracted the Fe(II) hologram using an equation similar to equation (6), and, although the reconstruction can be distinguished from the reconstruction obtained from the total [Fe(II) and Fe(III)], the quality of the results shows that it will be difficult to use the same approach on samples with larger and more complicated structures. Hosokawa *et al.* (2020 ▸) used a different approach to extract the Yb(II) hologram, where the Yb(II) hologram was recorded at an energy where only the Yb 2*p*_3/2_ electrons in Yb(II) were excited and emitted fluorescent X-rays. The reconstructions obtained showed the expected f.c.c. structure around Yb(III), whereas the reconstructed atomic image around Yb(II) showed large positional fluctuations. In principle, this would be a reasonable approach to obtain valence-sensitive holograms. However, for samples with low concentration of metal emitters, or for radiation-sensitive samples such as metalloproteins, it will be experimentally difficult to obtain valence-selective holograms using this approach.

## 5. Conclusions

In this work, we develop a novel approach for obtaining the local atomic structure around metallic clusters in protein crystals. By employing a 2D hybrid pixel detector to directly image the hologram patterns, a statistically significant hologram pattern was obtained in serial data acquisition mode, similar to those used in serial protein crystallography. The Mn *K*α hologram was recorded from PSII crystals prior to the onset of radiation-damage-induced reduction of the Mn_4_CaO_5_ clusters. The good agreement between the calculated and experimental hologram patterns and the analysis of the FS directions show that the recorded hologram pattern can be treated as a real-space projection of the atoms around the Mn emitters in the Mn_4_CaO_5_ clusters.

Furthermore, our new approach also allows valence-sensitive XFH experiments on protein crystals. Our preliminary results show that, by tuning the incident X-ray energy, the holograms from Mn(III) and Mn(IV) were extracted, and some distinct features observed in both the experimental and the calculated holograms allow some differentiation between them. Though additional data processing and more advanced reconstruction algorithms will be necessary to selectively reconstruct the local atomic structure around either Mn(III) or Mn(IV) from the holograms, the analysis of the FS patterns in the hologram, in combination with *R*-factor analysis, may allow the determination of which Mn sites each Mn ion occupies. This paves the way for future valence-sensitive XFH studies of other metal clusters in proteins and, considering that the valence states of these metal clusters play key roles in the functions of these proteins, there can be significant developments unlocked by further refinement of this approach.

## Figures and Tables

**Table 1:** Summary of the experimental conditions of the sXFH measurements on PSII

	Unit-cell parameters			Total photons collected (×10^6^)	
Sample	(Å)	(°)	No. of scan positions	At χ_H_, 6.565 keV	At χ_L_, 6.551 keV
001	*a* = 123.807, *b* = 228.591, *c* = 286.008	α = β = γ = 90	231	4.30	3.58
002	*a* = 125.444, *b* = 228.707, *c* = 286.563	α = β = γ = 90	144	3.17	2.59
003	*a* = 124.75, *b* = 229.485, *c* = 285.433	α = β = γ = 90	72	2.19	1.70
004	*a* = 124.234, *b* = 228.938, *c* = 285.951	α = β = γ = 90	80	2.22	1.65
005	*a* = 134.579, *b* = 227.073, *c* = 286.66	α = β = γ = 90	179	2.12	1.77
006	*a* = 121.072, *b* = 227.512, *c* = 286.063	α = β = γ = 90	138	1.76	1.43
007	*a* = 123.024, *b* = 228.847, *c* = 286.000	α = β = γ = 90	77	2.01	1.57
008	*a* = 124.049, *b* = 229.029, *c* = 290.245	α = β = γ = 90	143	4.64	3.74
009	*a* = 121.633, *b* = 228.184, *c* = 286.12	α = β = γ = 90	164	5.07	3.76

## References

[bb1] Ang, A. K. R., Marumi, R., Sato-Tomita, A., Kimura, K., Happo, N., Akagi, K., Sasaki, T. & Hayashi, K. (2021). *Phys. Rev. B*, **103**, 214106.

[bb2] Ang, A. K. R., Matsushita, T., Hashimoto, Y., Happo, N., Yamamoto, Y., Mizuguchi, M., Sato-Tomita, A., Shibayama, N., Sasaki, Y. C., Kimura, K., Taguchi, M., Daimon, H. & Hayashi, K. (2018). *Phys. Status Solidi B*, **255**, 1800100.

[bb3] Ang, A. K. R., Sato-Tomita, A., Shibayama, N., Umena, Y., Happo, N., Marumi, R., Kimura, K., Matsushita, T., Akagi, K., Sasaki, T., Sasaki, Y. C. & Hayashi, K. (2020). *Jpn. J. Appl. Phys.* **59**, 010505.

[bb4] Asada, M., Nagashima, H., Koua, F. H. M., Shen, J. R., Kawamori, A. & Mino, H. (2013). *Biochim. Biophys. Acta*, **1827**, 438–445. 10.1016/j.bbabio.2012.12.011 23313805

[bb5] Ballabriga, R., Campbell, M. & Llopart, X. (2018). *Nucl. Instrum. Methods Phys. Res. A*, **878**, 10–23.

[bb6] Barton, J. J. (1991). *Phys. Rev. Lett.* **67**, 3106–3109. 10.1103/PhysRevLett.67.3106 10044642

[bb7] Bortel, G., Faigel, G., Tegze, M. & Angelov, B. (2019). *J. Synchrotron Rad.* **26**, 170–174. 10.1107/S1600577518014686 30655482

[bb8] Bortel, G., Faigel, G., Tegze, M. & Chumakov, A. (2016). *J. Synchrotron Rad.* **23**, 214–218. 10.1107/S1600577515019037 26698066

[bb9] Chu, H. A., Hillier, W. & Debus, R. J. (2004). *Biochemistry*, **43**, 3152–3166. 10.1021/bi035915f 15023066

[bb10] Chu, H. A., Hillier, W., Law, N. A. & Babcock, G. T. (2001). *Biochim. Biophys. Acta*, **1503**, 69–82. 10.1016/s0005-2728(00)00216-4 11115625

[bb11] Cox, N., Rapatskiy, L., Su, J. H., Pantazis, D. A., Sugiura, M., Kulik, L., Dorlet, P., Rutherford, A. W., Neese, F., Boussac, A., Lubitz, W. & Messinger, J. (2011). *J. Am. Chem. Soc.* **133**, 3635–3648. 10.1021/ja110145v 21341708

[bb12] Dąbrowski, K. M., Dul, D. T., Roszczynialski, T. P. & Korecki, P. (2013). *Phys. Rev. B*, **87**, 064111.

[bb13] Daimon, H. (2018). *J. Phys. Soc. Jpn*, **87**, 061001.

[bb14] Daimon, H. (2020). *Jpn. J. Appl. Phys.* **59**, 010504.

[bb15] Dau, H., Grundmeier, A., Loja, P. & Haumann, M. (2008). *Philos. Trans. R. Soc. B*, **363**, 1237–1244. 10.1098/rstb.2007.2220 PMC2614103 17989002

[bb16] Dau, H. & Haumann, M. (2008). *Coord. Chem. Rev.* **252**, 273–295.

[bb17] Debus, R. J., Strickler, M. A., Walker, L. M. & Hillier, W. (2005). *Biochemistry*, **44**, 1367–1374. 10.1021/bi047558u 15683222

[bb18] Dul, D. T. & Korecki, P. (2012). *New J. Phys.* **14**, 113044.

[bb19] Fadley, C. S. & Len, P. M. (1996). *Nature*, **380**, 27–28.

[bb20] Faigel, G., Bortel, G. & Tegze, M. (2016). *Sci. Rep.* **6**, 22904. 10.1038/srep22904 PMC4786796 26965321

[bb21] Faigel, G. & Tegze, M. (1999). *Rep. Prog. Phys.* **62**, 355–393.

[bb22] Ferreira, K. N., Iverson, T. M., Maghlaoui, K., Barber, J. & Iwata, S. (2004). *Science*, **303**, 1831–1838. 10.1126/science.1093087 14764885

[bb23] Garman, E. F. (2010). *Acta Cryst.* D**66**, 339–351. 10.1107/S0907444910008656 PMC2852297 20382986

[bb24] Glöckner, C., Kern, J., Broser, M., Zouni, A., Yachandra, V. & Yano, J. (2013). *J. Biol. Chem.* **288**, 22607–22620. 10.1074/jbc.M113.476622 PMC3829347 23766513

[bb25] Guskov, A., Kern, J., Gabdulkhakov, A., Broser, M., Zouni, A. & Saenger, W. (2009). *Nat. Struct. Mol. Biol.* **16**, 334–342. 10.1038/nsmb.1559 19219048

[bb26] Hayashi, K., Happo, N. & Hosokawa, S. (2014). *J. Electron Spectrosc. Relat. Phenom.* **195**, 337–346.

[bb27] Hayashi, K., Happo, N., Hosokawa, S., Hu, W. & Matsushita, T. (2012). *J. Phys. Condens. Matter*, **24**, 093201. 10.1088/0953-8984/24/9/093201 22318258

[bb28] Hayashi, K. & Korecki, P. (2018). *J. Phys. Soc. Jpn*, **87**, 061003.

[bb30] Hosokawa, S., Happo, N., Hayashi, K., Kimura, K., Matsushita, T., Stellhorn, J. R., Mizumaki, M., Suzuki, M., Sato, H. & Hiraoka, K. (2020). *J. Phys. Soc. Jpn*, **89**, 034603.

[bb29] Hosokawa, S., Happo, N., Matsushita, T., Stellhorn, J. R., Kimura, K. & Hayashi, K. (2019). *Jpn. J. Appl. Phys.* **58**, 120601.

[bb31] Hosokawa, S., Happo, N., Ozaki, T., Ikemoto, H., Shishido, T. & Hayashi, K. (2013). *Phys. Rev. B*, **87**, 094104.

[bb500] Kabsch, W. (2010). *Acta Cryst.* D**66**, 125–132. 10.1107/S0907444909047337 PMC2815665 20124692

[bb32] Kamiya, N. & Shen, J.-R. (2003). *Proc. Natl Acad. Sci.* **100**, 98–103.

[bb33] Kang, L., Akagi, K., Hayashi, K. & Sasaki, T. (2017). *Phys. Rev. B*, **95**, 214106.

[bb34] Kawakami, K. & Shen, J. R. (2018). *Methods Enzymol.* **613**, 1–16. 10.1016/bs.mie.2018.10.002 30509462

[bb35] Kimura, K., Yamamoto, K., Hayashi, K., Tsutsui, S., Happo, N., Yamazoe, S., Miyazaki, H., Nakagami, S., Stellhorn, J. R., Hosokawa, S., Matsushita, T., Tajiri, H., Ang, A. K. R. & Nishino, Y. (2020). *Phys. Rev. B*, **101**, 024302.

[bb36] Kizaki, H., Hayashi, K., Lu, C., Happo, N., Hosokawa, S., Hidaka, S., Hayashi, S., Suzuki, M. & Uchitomi, N. (2022). *Phys. Rev. B*, **106**, 064434.

[bb37] Korecki, P. & Materlik, G. (2001). *Phys. Rev. Lett.* **86**, 2333–2336. 10.1103/PhysRevLett.86.2333 11289922

[bb38] Korecki, P., Novikov, D. V. & Tolkiehn, M. (2009). *Phys. Rev. B*, **80**, 014119.

[bb39] Korecki, P., Tolkiehn, M., Novikov, D. V., Materlik, G. & Szymonski, M. (2006). *Phys. Rev. B*, **74**, 184116. 10.1103/PhysRevLett.96.035502 16486723

[bb40] Kulik, L. V., Epel, B., Lubitz, W. & Messinger, J. (2007). *J. Am. Chem. Soc.* **129**, 13421–13435. 10.1021/ja071487f 17927172

[bb41] Kuznetsov, M. V., Ogorodnikov, I. I. & Vorokh, A. S. (2014). *Russ. Chem. Rev.* **83**, 13–37.

[bb42] Matsushita, T. (2018). *Phys. Status Solidi B*, **255**, 1800091.

[bb43] Matsushita, T., Muro, T., Matsui, F., Happo, N. & Hayashi, K. (2020). *Jpn. J. Appl. Phys.* **59**, 020502.

[bb44] Matsushita, T., Muro, T., Yokoya, T., Terashima, K., Kato, Y., Matsui, H., Maejima, N., Hashimoto, Y. & Matsui, F. (2020). *Phys. Status Solidi B*, **257**, 2000117.

[bb45] Plackett, R., Horswell, I., Gimenez, E. N., Marchal, J., Omar, D. & Tartoni, N. (2013). *J. Instrum.* **8**, C01038.

[bb46] Robblee, J. H., Cinco, R. M. & Yachandra, V. K. (2001). *Biochim. Biophys. Acta*, **1503**, 7–23. 10.1016/s0005-2728(00)00217-6 PMC3950273 11115621

[bb47] Roelofs, T. A., Liang, W., Latimer, M. J., Cinco, R. M., Rompel, A., Andrews, J. C., Sauer, K., Yachandra, V. K. & Klein, M. P. (1996). *Proc. Natl Acad. Sci. USA*, **93**, 3335–3340. 10.1073/pnas.93.8.3335 PMC39608 11607649

[bb48] Sano, K., Sasaki, T., Yoneyama, N. & Kobayashi, N. (2010). *Phys. Rev. Lett.* **104**, 217003. 10.1103/PhysRevLett.104.217003 20867129

[bb49] Sato-Tomita, A., Ang, A. K. R., Kimura, K., Marumi, R., Happo, N., Matsushita, T., Park, S., Shibayama, N., Sasaki, Y. C. & Hayashi, K. (2022). *Biochem. Biophys. Res. Commun.* **635**, 277–282. 10.1016/j.bbrc.2022.10.003 36308907

[bb50] Sato-Tomita, A., Shibayama, N., Happo, N., Kimura, K., Okabe, T., Matsushita, T., Park, S. Y., Sasaki, Y. C. & Hayashi, K. (2016). *Rev. Sci. Instrum.* **87**, 063707. 10.1063/1.4953453 27370459

[bb51] Shen, J. R. (2015). *Annu. Rev. Plant Biol.* **66**, 23–48. 10.1146/annurev-arplant-050312-120129 25746448

[bb52] Stellhorn, J. R., Hosokawa, S., Happo, N., Tajiri, H., Matsushita, T., Kaminaga, K., Fukumura, T., Hasegawa, T. & Hayashi, K. (2017). *J. Appl. Cryst.* **50**, 1583–1589.

[bb53] Stich, T. A., Yeagle, G. J., Service, R. J., Debus, R. J. & Britt, R. D. (2011). *Biochemistry*, **50**, 7390–7404. 10.1021/bi2010703 PMC3418060 21790179

[bb54] Suga, M., Akita, F., Hirata, K., Ueno, G., Murakami, H., Nakajima, Y., Shimizu, T., Yamashita, K., Yamamoto, M., Ago, H. & Shen, J. R. (2015). *Nature*, **517**, 99–103. 10.1038/nature13991 25470056

[bb55] Suzuki, M., Kawamura, N., Mizumaki, M., Terada, Y., Uruga, T., Fujiwara, A., Yamazaki, H., Yumoto, H., Koyama, T., Senba, Y., Takeuchi, T., Ohashi, H., Nariyama, N., Takeshita, K., Kimura, H., Matsushita, T., Furukawa, Y., Ohata, T., Kondo, Y., Ariake, J., Richter, J., Fons, P., Sekizawa, O., Ishiguro, N., Tada, M., Goto, S., Yamamoto, M., Takata, M. & Ishikawa, T. (2013). *J. Phys. Conf. Ser.* **430**, 012017.

[bb56] Tanaka, A., Fukushima, Y. & Kamiya, N. (2017). *J. Am. Chem. Soc.* **139**, 1718–1721. 10.1021/jacs.6b09666 28102667

[bb57] Tegze, M. & Faigel, G. (1996). *Nature*, **380**, 49–51.

[bb58] Umena, Y., Kawakami, K., Shen, J. R. & Kamiya, N. (2011). *Nature*, **473**, 55–60. 10.1038/nature09913 21499260

[bb59] Yachandra, V. K., Sauer, K. & Klein, M. P. (1996). *Chem. Rev.* **96**, 2927–2950. 10.1021/cr950052k 11848846

[bb60] Yano, J., Kern, J., Irrgang, K.-D., Latimer, M. J., Bergmann, U., Glatzel, P., Pushkar, Y., Biesiadka, J., Loll, B., Sauer, K., Messinger, J., Zouni, A. & Yachandra, V. K. (2005). *Proc. Natl Acad. Sci.* **102**, 12047–12052. 10.1073/pnas.0505207102 PMC1186027 16103362

[bb61] Yano, J., Pushkar, Y., Glatzel, P., Lewis, A., Sauer, K., Messinger, J., Bergmann, U. & Yachandra, V. (2005). *J. Am. Chem. Soc.* **127**, 14974–14975. 10.1021/ja054873a PMC3981608 16248606

[bb62] Zeldin, O. B., Gerstel, M. & Garman, E. F. (2013). *J. Appl. Cryst.* **46**, 1225–1230.

[bb63] Zouni, A., Witt, H. T., Kern, J., Fromme, P., Krauss, N., Saenger, W. & Orth, P. (2001). *Nature*, **409**, 739–743. 10.1038/35055589 11217865

